# How to design effective educational videos for teaching evidence-based medicine to undergraduate learners – systematic review with complementing qualitative research to develop a practicable guide

**DOI:** 10.1080/10872981.2024.2339569

**Published:** 2024-04-14

**Authors:** Lukas Niekrenz, Cord Spreckelsen

**Affiliations:** a Institute of Medical Informatics, University Hospital Aachen, RWTH Aachen University, Aachen, Germany; b Institute of Medical Statistics, Computer and Data Sciences, Jena University Hospital, Friedrich Schiller University Jena, Jena, Germany

**Keywords:** Video-based eLearning, asynchronous learning, adaptive requirement analysis, educational video design, elearning design, guidelines as topic

## Abstract

**Background:**

eLearning can be an effective tool to achieve learning objectives. It facilitates asynchronous distance learning, increasing flexibility for learners and instructors. In this context, the high educational value of videos provides an invaluable primary component for longitudinal digital curricula, especially for maintaining knowledge on otherwise rarely taught subjects. Although literature concerning eLearning evaluation exists, research comprehensively describing how to design effective educational videos is lacking. In particular, studies on the requirements and design goals of educational videos need to be complemented by qualitative research using grounded theory methodology.

**Methods:**

Due to the paucity of randomized controlled trials in this area, there is an urgent need to generate recommendations based on a broader fundament than a literature search alone. Thus, the authors have employed grounded theory as a guiding framework, augmented by Mayring’s qualitative content analysis and commonly used standards. An adaptive approach was conducted based on a literature search and qualitative semi-structured interviews. Drawing on these results, the authors elaborated a guide for creating effective educational videos.

**Results:**

The authors identified 40 effective or presumedly effective factors fostering the success of video-based eLearning in teaching evidence-based medicine, providing a ready-to-use checklist. The information collected via the interviews supported and enriched much of the advice found in the literature.

**Discussion:**

To the authors’ knowledge, this type of comprehensive guide for video-based eLearning needs has not previously been published. The interviews considerably contributed to the results. Due to the grounded theory-based approach, in particular, consensus was achieved without the presence of a formal expert panel. Although the guide was created with a focus on teaching evidence-based medicine, due to the general study selection process and research approach, the recommendations are applicable to a wide range of subjects in medical education where the teaching aim is to impart conceptual knowledge.

## Introduction

### Motivation

Despite extensive efforts to establish effective teaching in the fields of medical statistics and epidemiology, we observed the persistent problem of ‘statistical illiteracy,’ that is, the inability to adequately interpret statistical data and test results, which is known to be an issue in medical risk communication and decision-making [1]. Although most medical curricula, as at our institution, address medical statistics and epidemiology quite extensively, leading to satisfactory exam results, the presence of statistical illiteracy in trained doctors points to a lack of long-term retention [1,2].

Longitudinal learning, including spaced learning, promises longer recall of imparted knowledge, especially when active learning techniques (e.g., quizzes) are employed, while the feasibility of longitudinal educational modules is dependent on whether asynchronous learning opportunities are implementable at reasonable costs. With this in mind, we have focused our research on video-based eLearning, which we consider to be a suitable tool for this use case.

In teaching evidence-based medicine, we have experienced an urgent need for the teaching of conceptual knowledge and the communication of ideas, concepts, and techniques for problem solving. This applies even more in our use case as the ‘statistical’ way of thinking is imported into medical school curricula from a different field. To ensure long-term retention of this way of thinking, short, easy-to-understand chunks of knowledge seem to be ideal. This becomes even more important if the statistical way of thinking is not integrated into learners’ daily lives. In particular, the understanding of unaccustomed concepts may depend on opportunities for review. This is another reason for focusing on video-based eLearning to support these curricular components in medical education.

Motivated by the above considerations, we implemented a longitudinal, asynchronous digital curriculum based on educational videos at RWTH Aachen’s faculty of medicine to supplement existing classroom teaching. Educational video is a cornerstone of our longitudinal curriculum and is augmented by several other modalities (e.g., text-based learning materials, e-tests, and player-vs.-player quizzes). Therefore, a substantial need exists for videos that generate concrete learning achievements.

In our search for a comprehensive, ready-to-use guide for creating video-based eLearning offerings in medical education, we were confronted with the absence of such work. For this reason, we decided to conduct a systematic review and interviews to define best practices and to create such a guide.

### State of research

Prior to describing the creation of a comprehensive guide on video-based eLearning, a brief overview of the known issues should be helpful.

Studies have shown that eLearning in medical education is effective for imparting knowledge under specified conditions, for example, in teaching evidence-based medicine [3,4]. However, eLearning is not always more effective than other forms of learning [5]. Vaona et al. stated in their Cochrane Review that eLearning interventions cause a large positive effect when compared to no intervention, and only a small positive effect when compared to traditional learning, although these results are not conclusive. They concluded, ‘Even if e‐learning could be more successful than traditional learning in particular medical education settings, general claims of it as inherently more effective than traditional learning may be misleading’ [5].

With regard to videos, the literature shows that they can be useful for their impact on learning [6–8] and are widely used in education [9]. A high educational value is also attributed to videos because of their advanced multimedia level in Mayer’s cognitive theory of multimedia learning [10,11]. This theory is well known and widely respected in the literature for its assumption that different channels process visuals and auditive content in the learner’s mind, with resulting implications that are outlined in more depth below. Consequently, some authors even describe this theory as unique with respect to its impact on multimedia design [12].

Moreover, students’ perceptions of educational videos are often subjectively reported to be positive in various contexts, not just at our institution [13,14], and videos may be more engaging than conventional learning materials (e.g., textbooks) [8]. Nonetheless, Guo et al. have shown that student engagement collapses after a median of six minutes of video [9]; therefore, we assume that there is either a pressing need for short videos or for the improvement of educational video formats.

Furthermore, some studies show that educational video does not robustly lead to better knowledge acquisition or effectiveness compared to other forms of learning [15–17], although, as with eLearning in general, no comprehensive real-world assessment has been provided. This insight might depend on how videos are designed and the context in which they are used [5,17]. Unfortunately, researchers do not always report how the videos or eLearning content used in evaluation trials were created; consequently, the knowledge acquired to develop a best-practice video-based eLearning program is limited [18]. However, since many eLearning modalities include educational videos (e.g., [19]), our focus on best practices for educational video has the potential to make a highly pertinent contribution toward better eLearning.

### Instructional design for video-based eLearning

Our search for guidance on effective eLearning design yielded general underlying principles, such as the ADDIE model, which is based on generic analysis, design, development, implementation, and evaluation [20]. Its application helps to establish a structure for creating and maintaining educational interventions but does not answer the question of how exactly the individual domains should be implemented.

Guidelines for instructional design and their possible implementation by leveraging software are found in Overbaugh’s ‘Research-Based Guidelines for Computer-Based Instruction Development,’ published in 1994 [21]; however, this publication neglects the current developments and possibilities of educational videos.

In the design of effective educational videos, Mayer’s cognitive theory of multimedia learning [10,11] represents a milestone publication. According to this theory, the learner’s mind uses different ‘channels’ to process verbal and visual content (‘dual channel’), and each channel is only able to handle a limited amount of information (‘limited capacity’). In addition, learning success hinges on the learner’s cognitive processing during learning (‘active processing’) [10]. This theory is consistent with and incorporates the cognitive load theory of Sweller et al., which assumes that the verbal and visual channels have respective limited capacities [22,23]. Although the principles introduced by Mayer are essential for creating well-designed videos, applying them in isolation does not necessarily lead to perfect results, as other design and instructional aspects remain unaddressed.

Furthermore, various studies on effective educational video design exist [14,24–28], some of which were published only after our project began [29,30]; however, we consider these valuable publications to be insufficient on their own to provide a comprehensive guide to video-based eLearning or to meet our needs in teaching evidence-based medicine due to their focus on specific aspects, different levels of conceptions, or experience-led approaches rather than on a comprehensive, ready-to-use, evidence-based feature set or framework for generating effective educational videos.

As we ourselves would have greatly appreciated evidence-based guidelines for creating instructional videos for our own and other institutions that explained related difficulties (or at least a guiding checklist pointing out important issues traditionally encountered), especially in creating or evaluating eLearning resources [31], we decided to strive for such a guiding document.

To ensure the highest possible quality for this document, a systematic review, which ideally can include several randomized controlled trials per item, suggests itself. To close any gaps in the evidence in a reasonable way, a consensus should then be reached, as in a formal guideline-creation process. However, after a preliminary search, we encountered a paucity of randomized trials in this field. To overcome this literature gap under the given circumstances, an approach along the lines of grounded theory [32–34] was deemed particularly appropriate in view of the topicality and significance of the subject. A grounded theory approach can augment existing evidence from the literature at a higher level than expert opinions alone to achieve categorization, relativization, and consensus for a guiding document. This eliminates the necessity of conducting the multitude of studies required while still achieving high representativity based on a defined methodology.

### Objective

In the belief that tailoring an educational program to a target group may be useful to maximize student engagement and achievement, we investigated our learners’ needs by focusing our research on this question: *What form should successful video eLearning take, especially in teaching evidence-based medicine?*

## Materials and methods

**Figure 1.**
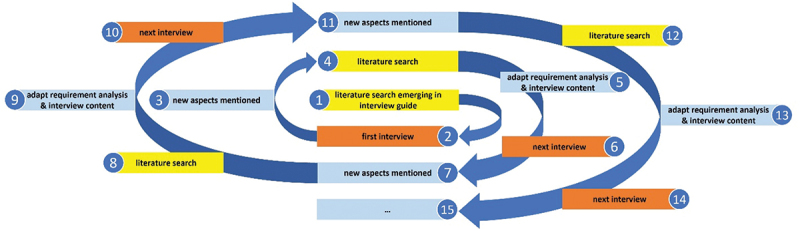
“Spiral model” – Process model for adapting the requirement analysis to subsequent interviews.

To establish requirements for educational video (general criteria for adequate use and specific design requirements), we dovetailed the literature search and interviews after conducting preliminary requirement analysis based on the literature, resulting in the ‘spiral model’ used in this study. Our approach is summarized in Figure 1.

To better interpret, balance, and apply the findings in the literature (which were often obtained under narrow parameters) as well as theories, we employed the following methodology:

We gathered evidenced insights from the literature; however, to consistently interpret and merge the findings, we required qualitative research methods and statements from different stakeholder perspectives. In addition to providing a methodologically independent confirmation of previous findings, this approach allows the discussion of aspects not yet considered in the literature.

**Figure 2.**
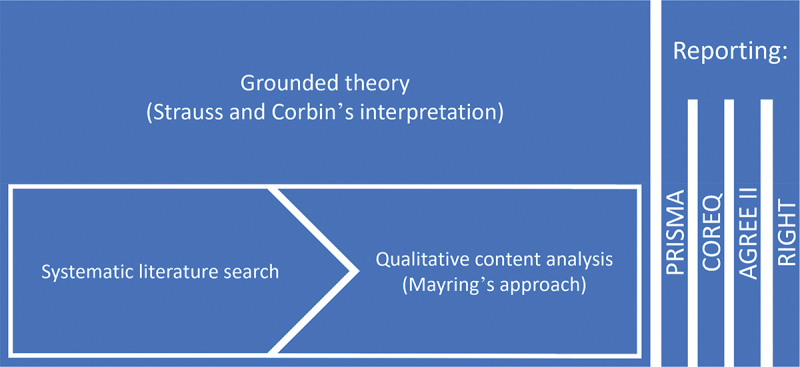
Grounded theory approach incorporating different standards of reporting.

To create a defined methodology for our work, we selected grounded theory as the guiding framework and incorporated commonly used standards to strengthen the methodology and meet established reporting standards, as shown in Figure 2.

To this end, we used grounded theory methodology per Strauss and Corbin’s interpretation [32–34]. Furthermore, we respected Mayring’s approach to qualitative content analysis [35,36] in planning, performing coding, interpretation, evaluation, and reporting.

Our research approach is based on essential elements of grounded theory. These include the analysis of data obtained, which is directly interwoven with data collection, whereupon the latter is influenced in the sequel. Furthermore, regarding ‘theoretical sampling,’ we decided to make our interview population as diverse as possible to obtain conceptual representativeness for the stakeholders, especially regarding study progress, age, and academic performance. In addition, we anchored the aspect of ‘theoretical saturation’ in our approach, terminating the recruitment of further interview partners once saturation was reached.

However, our objective of creating a comprehensive guide for successful video eLearning involves various aspects that we do not believe, in good conscience, would be limited to a few core categories that are axially related to the remaining categories; therefore, we deviated from the established principles here.

The subsequent development of the interview content by applying grounded theory methodology enabled the achievement of categorization, relativization, and consensus on the part of the interviewees, who represented the respective stakeholders at the end-user level. To achieve the highest possible quality, our work was guided by the characteristics required by the RIGHT statement [37,38] and in AGREE II [39–41], which are commonly used for clinical guideline development and recommended by the EQUATOR network. It should be noted that we are aware that our work does not fully reflect a ‘classical’ guideline development approach with an expert panel.

To comply with established standards in the individual steps of our work, we followed the PRISMA checklist [42] for the literature search underlying our grounded theory approach and the COREQ statement [43] for reporting qualitative characteristics. Since human beings were involved in our study, we consulted the Ethics Committee at University Hospital RWTH Aachen. They approved our protocols, declaring that their vote is not required (ID EK 091/18).

### Literature search

We performed a literature search using PubMed due to our focus on medical education. The search began in November 2017 and was intensively conducted from February – March 2018, as well as subsequently (see below) to stay up to date. We chose an explorative approach to generate a basis for semi-structured interviews and fulfill the requirement analysis.

We searched for literature describing concepts and methods for designing eLearning content, especially videos or parts thereof (e.g., animations). We focused our search on studies involving undergraduate students as the primary population and graduate learners as the secondary population, each trained on concepts and theories using different design elements, which were then compared to each other or to traditional design elements, allowing evidence-based principles to be derived. We subsequently defined the eligibility criteria. We included articles that presented theories and studies on how to design video-based learning or eLearning for concept delivery, giving concrete (evidence-based) advice. We included reviews, randomized controlled trials, controlled trials, case reports, and expert opinions. Articles were excluded from this synthesis if they did not primarily focus on asynchronous learning capabilities or mainly discussed concepts for flipped classrooms, virtual seminars, or virtual enriched group work (e.g., problem-oriented learning); if they focused on procedural craft skills as often taught in surgery (the wrong setting); or if they only described the status quo of eLearning applications without offering critical appraisal or deriving advice. Because of our focus on video-based, asynchronous eLearning, we limited the search to the period starting in 2006, the first full year since the emergence of YouTube® as a well-known online tool for making videos available online.

We started our search using ‘((((video OR educational video OR video-based eLearning OR video based learning OR video eLearning OR video learning OR medical video OR video tape recording[MeSH]) AND (online)) AND (medical students[MeSH] OR students[MeSH])) AND (educational technology[MeSH])) AND (guidelines as topic[MeSH])’ as the initial search string. After obtaining only one relevant paper out of two results, we widened our search by varying the search string by combining the terms in the initial search string in different ways. Our search strings can be found in Box 1 in the Appendix.

**Figure 3.**
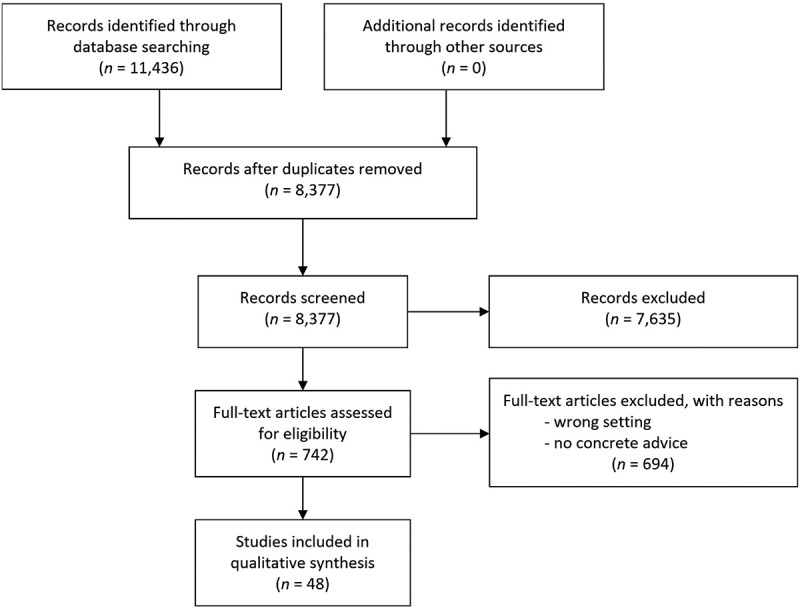
Graphical summary according to the PRISMA guidelines.

The titles and abstracts were screened for potentially relevant articles, and then full texts were accessed. Figure 3 presents a graphical summary according to the PRISMA checklist [42].

### Preliminary requirement analysis

Based on the literature search described, we conducted a preliminary requirement analysis and expanded it according to assumptions arrived at deductively and inductively. User stories were chosen as the requirement analysis format. Based on this analysis, we selected important and controversial points (e.g., style of animations, Khan style [handwritten, step-by-step explanations with voice commentary], examples in explanations [amount and quality], and interactions) to serve as interview topics and developed a semi-structured interview guide. To avoid focusing solely on the points revealed by the literature search, the interview guide included open and broad questions on how eLearning and educational video should be developed in the participant’s opinion.

This interview guide can be found in the Appendix.

### Adapting the data elicitation – “spiral model”

To enrich and adapt the preliminary requirement analysis, we immediately took the new aspects mentioned in an interview into consideration for discussion with participants in subsequent interviews.

For this purpose, a literature search followed every interview in which new aspects were mentioned. Based on the results and the interview content itself, we enhanced the preliminary requirement analysis and adapted the interview content for subsequent interviews, as shown in Figure 1. Furthermore, categorization, relativization, and consensus were achieved via this process, as the points mentioned in previous interviews were thereby discussed with the interviewees. Consequently, interviewees were confronted with either contrasting or similar statements previously made by others, in addition to statements derivedfrom literature, and then provided their statement.

### Subsequent search

To stay up to date, we conducted annual literature searches using our initial search string and the first four strings derived therefrom (see *Box 1* in the Appendix) to add new articles and aspects. The following outlines our suggested updating procedure:

A renewed literature search should be conducted at least every three years and new findings added. In the event of substantial changes or the emergence of new features considered worthy of discussion, a renewed implementation of the interview-based methodology should be considered at least every 10 years.

### COREQ-compliant description of the interview process

To meet the reporting standards in qualitative research, we respected the consolidated criteria for reporting qualitative research (COREQ) by Tong et al. [43].

### Study design

As described above, the interview content was defined based on the preliminary requirement analysis; however, performing the interviews successively resulted in the expansion of the preliminary requirement analysis and, therefore, the interview content.

We selected the participants by academic semester. At least one participant from each year was recruited to obtain a cross section of the various potential eLearning needs of students across several years. The main target group of our eLearning curriculum consisted of students for whom the start of research activities and clinical work were assumed to be imminent. In addition, without such eLearning, students in advanced semesters would have had their last training in evidence-based medicine years before. Therefore, we aimed to recruit a disproportionate number of 4th year students (according to the German six-year curriculum) as representatives of the main target group.

Other factors for recruiting participants were cross sections of academic performance and age, the approximate female/male ratio by course of study, and reachability by the interviewer.

### Data collection

We provided a semi-structured interview guide for every interview (see the Appendix). We conducted only one interview per participant. After approval, the interviews were audio-recorded using a professional pocket music recorder. No participant disagreed to the recording. In addition, field notes were taken during and immediately after the interviews.

Following the concept of ‘theoretical saturation’ from grounded theory, we planned to cease further interviews when the last interview conducted did not add new relevant information. To ensure a sufficiently large cross-section, the minimum number of participants was set at 11 following the considerations stated above.

The transcripts were not returned to the participants as our research variant was based on Mayring’s qualitative content analysis approach and grounded theory methodology and, thus, complete transcripts were not created.

### Analysis

Our analysis was guided by this research question: *What form should successful video eLearning take, especially in teaching evidence-based medicine?* Due to our preliminary requirement analysis, we documented our preconceptions in our interview guide. We chose a mixed research design incorporating descriptive (deductive category development, top-down process) and explorative (inductive category development, bottom-up process) elements [35] to investigate our learners’ needs and desires and to discuss specific points derived from the literature. As a coding unit of up to three keywords (in the sense of a paraphrase) was defined, the context unit comprised up to several contiguous sentences. The evaluation unit consisted of interviews. Thus, all statements concerning a single category were analyzed consecutively.

To save time and resources, we transcribed the audio-recorded interviews only partly in the spirit of a selective protocol [35]. Due to limited resources, the audio material was transcribed by the researchers themselves. However, this allowed the transcription and coding rules to be applied simultaneously. Whenever a passage met the requirements of the coding guide for deductive categories (available in the Appendix) or our procedure for inductive category development (see below), the entire passage was transcribed and associated with the interview number and the time code that marked the start of the relevant passage in the audio file. Therefore, the partly transcribed interviews nevertheless included relevant information.

To ensure that the inductive category assignment complied with the open questions and possible non-predetermined interview content, we referred to our research question and previous content-analytical units. We established the categories as every implementable feature, design element, or variant of content presentation that could be considered a (potentially) valuable part of a best-practice eLearning implementation. As a level of abstraction, we defined generally applicable statements, comments, and viewpoints from which advice could be derived and which could be enriched with concrete examples and statements, although the latter was not necessary.

After the first run of coding, the categories were controlled, and interrelated categories were joined. During the second instance of listening to all the interviews, the complete category list was accessible. Aspects mentioned regarding each category were allocated and transcribed as before, including the assignment of time stamps. For the analysis, we used transcribed passages. The time stamps ensured that they were reidentifiable in the raw data. We adapted the categories during coding.

Intercoder agreement was ensured by weekly supervision dialogues between the authors, including the presentation, discussion, and reworking of the coding performed by confirming the analysis, as suggested by Mayring for very open material with an explorative research question [35]. In addition, as the project progressed, we discussed the theories and findings of the study at department meetings to obtain surrogate communicative validation from our colleagues as learners and educators.

### Formulation of recommendations

To meet our objective of providing comprehensive guidelines for creating educational videos despite the paucity of randomized controlled trials, we have included as recommendations all characteristics shown or assumed to be effective, provided that the following criteria apply:

– the derived recommendation appears relevant for concept delivery to the target group (undergraduate learners);

AND

– the derived recommendation is based on a higher level of evidence than qualified expert opinion, OR, if it is an expert opinion, it was confirmed in discourse with the interviewees AND/OR the authors. Furthermore, we made the decision to allow the formulation of recommendations based on the applicable legal situation and the advice derived therefrom.

## Results

Systematic research yielded no paper describing a comprehensive evidence-based best practice approach to educational video creation for teaching evidence-based medicine. However, we found papers describing approaches to creating videos in other fields of medicine [29,44] and scientific education [45] as well as general considerations regarding various aspects of creating videos [30,46] or eLearning/computer-based learning materials and learning platforms [21,47].

Like many previous researchers, we agree that Mayer’s cognitive theory of multimedia learning is an important underpinning concept for creating educational video content. Much advice on creating well-designed educational videos can be traced back to his principles of ‘dual channels,’ ‘limited capacity,’ and ‘active processing’ [29], including achieving the balanced utilization of both channels while avoiding cognitive overload to provide the best possible processing of content as efficiently as feasible. In fact, the appropriate amount of information on each channel is a key factor for well-designed learning materials.

Furthermore, research by de Leeuw et al. describes a model for designing postgraduate digital education, mentioning important points for creating, maintaining, and evaluating eLearning offerings on a conceptual level; however, no concrete advice is given on how to design the educational intervention itself [48].

We also identified many additional points, such as design, administrative, and legal (i.e., data protection, copyright, and personality rights) issues.

Table 1 presents the detailed results of our spiral model approach, including the literature search and the interviews, while Table 2 shows the results of the interview analysis.

**Table 1.** Literature-based requirement analysis for eLearning content and educational videos – User stories.

Keyword/Topic	As a …	I want …	so that …	Example/Advice/Note	Reference(s)	Approved by interviews
Dual channel/Manage processing/Synchronizing/Matching	student	to get videos	I can follow the content as well visually as auditorily and build connections between audio and video		Mayer [10] Mayer & Moreno [22] Brame [24]	++
Continuous input flow	student	animations, which are building up, to be in the videos	I get a continuous input flow	You may also consider the ‘Khan style’.	Mayer [10] Mayer & Moreno [22] Guo et al. [9]	++
Khan style	creator	to use the Khan style (hand-written step-by-step explanation with voice commentary)	the students are more engaged due to the equal levels of the student and teacher	CAVE: controversial!	Guo et al. [9] Cross et al. [49]	0/+
Good visuals	student	to see well-made visuals	I can learn more effectively with them	Try to create clean and timeless visuals.	Norman [26] Iorio-Morin et al. [28] Choe et al. [30]	(++)
Reduce extraneous processing	student	the videos to be ‘clean,’ without distracting material	I concentrate on the learning objectives	no background music, etc.; controversial, especially in teaching procedures without additional text	Mayer [10] Wong [50]	++
− Weeding	creator	to plan for the format	the video does not contain unnecessary information and can be produced faster	CAVE: unnecessary vs. interesting and vivid detail → see Personalization	Guo et al. [9] Overbaugh [21] Brame [24] Norman [26]	
− Avoid Redundancy	student	to see content presented with animations and not see the spoken text	I am focused on the subject matter and do not read what I am hearing		Mayer [10]	
− Signaling	student	to be taken to the very important points	I can focus on them	Highlight the main points.	Mayer [10] Brame [24]	
− Temporal continuity	student	to see the animation/keyword while it is verbally explained	I can process the content simultaneously on both channels	Present visual and audio content at the same time.	Mayer [10]	
− Spatial continuity	student	to see corresponding text and animation near each other	I can focus on one part of the screen	Present corresponding material in synchrony.	Mayer [10]	
Manage essential processing/Learner control − Segmenting	student	to work with structured/divided videos	I learn from short chapters that I can process	Use checkpoints after short parts of your video, which must be clicked on.	Mayer [10] Overbaugh [21]	++
− Use of chapters	student	to get videos using chapters	I can navigate directly to the relevant chapters I want to repeat		Roshier et al. [14] Brame [24]	++
− Limit video length	student	to get short (roughly six-minute-long) videos	I watch the video up to its end	Mind the length while scripting; longer videos may be useful, too.	Guo et al. [9] Dong & Goh [25] Young et al. [29]	0/+
– Pre-training	student	to get taught/an introduction prior to a video curriculum	I can follow more easily (e.g., because I know the technical terms)		Mayer [10] Overbaugh [21]	
Personalization	student	to be addressed in a conversational style and/or informal setting	I can build a social partnership and try harder to understand the topic	Narrate the lesson from your perspective. Show your interest in the topic. You may consider the inclusion of interesting details that are not directly linked to the learning goals.	Guo et al. [9] Mayer [10] Brame [24] Overbaugh [21] Maloy et al. [51]	
− Optimizing the voiceover	student	the narration to have simple syntax	I can follow the speaker easily and effectively	Do not worry about an excessively simple script.	Iorio-Morin et al. [28]	
− Speed	student	to get videos in the appropriate speed	I can follow easily and effectively	Suggestions: ≥ 160 words per minute (wpm) [9]; 185–254 wpm [24]	Guo et al. [9] Brame [24]	++
− Target group	creator	to match the content for the target group	the students are more engaged	How can you show the students that it is content for *them*; you may contextualize your videos.	Brame [24] Fleming [46]	
− Pattern	creator	to develop and use a recognition pattern (at the beginning of each video)	the videos become more structured	Think of structuring your content, and develop a welcome pattern.	Norman [26]	
− Humor	student	to see humorous videos	I learn better and am more motivated	Be wary of excessive humor but consider light humor to break the ice.	Iorio-Morin et al. [28]	0/+
− Talking head	student	to see the speaker’s face at least occasionally	I am more engaged although it might be more distracting	CAVE: controversial!	Guo et al. [9] Dong & Goh [25]	0/+
Guiding question	student	to get focused on the relevant topics through guiding questions	my interest becomes aroused, I know what the lesson’s goal is and am less distracted	Think of practical, interesting, or provocative questions to start your lesson.	Overbaugh [21] Brame [24] Norman [26]	
Active Learning/Questions/Repeated testing	student	to be asked questions on the topics repeatedly during or after the videos	I internalize the messages of the videos		Spreckelsen & Juenger [52] Brame [24] Overbaugh [21]	++
Games	student	to be able to participate in a quiz with my course mates or to play games concerning the learning content	I am more engaged and learn while playing	Quizzes can be implemented easily if you have a pool of MC questions.	McCoy et al. [53]	0/+
Modularity	creator	to create modular videos	I can adapt these to other contexts	Think about possible fields of use before creating content.	Norman [26]	
	creator	to create self-contained learning entities	I can use them in different courses and over a long time	Avoid links, etc., to outside resources in your videos; do not number videos so that you can combine them in new ways.	Norman [26]	
Context	creator	to embed the videos in the (medical) context	the students are more motivated learners because they imagine the topics applied in context	Tell your students why they should learn the content.	Overbaugh [21] Brame [24]	(++)
Examples	student	to be confronted with relevant (medical) examples and examples that I understand intuitively (e.g., examples from everyday life)	I am more enthusiastic concerning the learning content	Think about your students’ knowledge and choose examples from prior courses; deliver an additional point of view to support understanding; think about relevant stories and narrations.	Overbaugh [21] Malloy et al. [51] Adam et al. [54]	++
Appropriate content	creator	to choose appropriate content	content is delivered that benefits from the ‘video’ presentation mode	Concepts can be delivered well via videos.	Rana et al. [27] Iorio-Morin et al. [28]	
Take-home messages	student	the videos to end with take-home messages	I can remember the relevant points better		Phelan [55]	++
Usability	student	to use an eLearning platform that simply works and is visually appealing	I can concentrate on the learning content and am engaged in using this learning modality		Roshier et al. [14]	++
– Mobile use/Screen management	student	the videos on a platform that is enabled for mobile use/the screen content is well managed	I can learn on any device I want at any place	Make your videos enjoyable on small screens (e.g., 4.7”).	Jang & Kim [56] Choe et al. [30]	++
− File format	student	to use the videos on any device ‘out of the box’	I do not face barriers to using the videos	Use common formats that most operating systems support (e.g., mp4/HTML5).	Dong & Goh [25]	
− Availability	student	to get access to the contents early	I can prepare my course any time I want	The content should ideally be completely produced before the course starts.	Margolis et al. [57]	(++)
Planning in advance	creator	to plan the videos	I can produce high-quality video content and identify suboptimal shoots		Guo et al. [9] Iorio-Morin et al. [28]	
− Scripting	creator	to script out the video	I can produce the video more effectively, and I can easily create subtitles		Norman [26]	
	creator	to script out the video	I can create a summary for the students		Norman [26]	
Administrative − Monitoring	teacher	to be able to monitor the use of the eLearning content	I can remind tardy students; I can identify topics that may still be challenging	CAVE: Depending on your institution’s eLearning privacy policy, metadata may not always be available by default.	Rana et al. [27]	
	creator	to be able to monitor the use of the eLearning content	I can evaluate how videos were used to identify attributes that are associated with viewing characteristics	Metadata could be a valuable indicator of the acceptance of, say, design implementations.	Rana et al. [27]	
	scientist	to be able to monitor the use and test results of the eLearning content	I can evaluate the learning outcomes aligned with using characteristics	CAVE: Be aware of data protection laws while planning your studies.		
– Legal & Licenses	creator	to use only content that I am allowed to use	I can provide the videos	Try to create content on your own as much as possible so that you hold all rights to it.	Roshier et al. [14]	
− Evaluation and Review	creator	my videos to be reviewed and evaluated in a preplanned manner	I can provide even better content	Establish review structures and procedures; plan an evaluation survey at the end of the course and provide a feedback e-mail address.	Roshier et al. [14] Young et al. [29] Authement & Dormire [47] Yavner et al. [58]	

Notes. ++: interviews support statement strongly; 0/+: results of interviews were not consistent, but a tendency could be found; (++): content of the interviews supports statement indirectly/implicitly; a shaded row means the category was defined deductively.

**Table 2.** Results of analysis of semi-structured interviews at RWTH Aachen University in 2018 (*n* = 11).

Keyword/ Topic	Leading quotation(s)	Number of supporting statements	Conclusion	Comments/ Additional information
Testing	‘You did something actively and did not just read things through, so you were constantly required to perform and I think you then simply learned better’ [ID01].	9 supporting 1 neutral 0 contradicting	Implement testing.	interviewees’ requests: − no excessive workload − not too redundant − appropriate difficulty (not too easy) − provide solutions
Khan style	‘It’s not bad to see things written’ [ID02].	6 supporting 1 ambivalent 1 contradicting	Implement subsequent animations/ writing if possible, and check traditional Khan style for suitability in individual cases.	Note: The content of the interviews changed from traditional handwritten Khan style to subsequent animations.
Animations	‘It’s cool for schematics if they build up one after the other’ [ID03].	5 supporting 0 contradicting	Implement subsequent animations if possible.	
Usability	‘[It’s important] that it works logically and simply somehow. The easier, the better’ [ID03].	7 supporting 0 contradicting	Try to use a simple and intuitive eLearning environment.	Depending on which software is used at your institution for students’ eLearning, you may use this familiar software.
Mobile use	‘[…] especially when it’s also possible on my mobile phone’ [ID01].	7 supporting 0 contradicting	Make the videos available on mobile devices.	
Structuring	‘Do I find that under “human medicine,” under “lecture,” under “media library,” or under “no idea”?’ [ID03]. ‘I rarely use [our institution’s media library] because it’s too confusing’ [ID02]. ‘The titles have to be well chosen so that they are concise and describe the content’ [ID04].	3 supporting 0 contradicting	Give your eLearning environment a good structure.	You may also tag your videos/content and implement a search function.
Chapters	‘[I’d appreciate] it if the videos were broken down to sub-items so that you would only have to look at the sections you wanted to see’ [ID05].	6 supporting 0 contradicting	Implement chapters.	
Length	‘The maximum would be about 15 minutes because, beyond then, I would no longer be attentive. Five minutes is already a good length, but the videos must not be shorter. Something between 5 and 15 minutes’ [ID05]. ‘Using chapters, the length is not so important anymore’ [ID06].	8 supporting 0 contradicting	Try to create short videos, but do not worry if you exceed the six-minute limit slightly.	
Speed	‘Not too fast and not too slowly’ [ID06].	4 supporting 0 contradicting	Extract ideal speed from literature.	Note: Speed was considered an important success factor for educational videos.
Take-home messages	‘I think that’s actually quite good because you learn the beginning and the end well’ [ID06].	5 supporting 0 contradicting	Formulate take-home messages.	
Reduce extraneous processing	‘There was so much background information – that was too much of a good thing’ [ID07].	6 supporting 0 contradicting	Avoid too much background information; reduce distractions (content-wise & artistically).	Note: Nevertheless, the topic should be covered completely.
Talking head	‘I’d find that more personal’ [ID08]. ‘I don’t care to see the speaker – it can be good, but it doesn’t have to be’ [ID02]. ‘I’m not a fan of [educational] videos in which you see the speaker. I don’t like that at all’ [ID01].	2 supporting 3 supporting 0 supporting	Check the talking head for suitability in individual cases. Ensure that the talking head does not interfere with the display of other elements.	You may consider both weaning on the one hand and more engagement and personalized videos on the other. Note: Many participants expressed concern about the space needed on the screen.
Medical examples vs. everyday-life examples	‘[I’d appreciate] both; first, I would look at everyday life and then at what helps me later in my job’ [ID04]. ‘I thought it was good that they were medical tests. […] But it should already be realistic’ [ID09]. ‘I actually always prefer everyday examples’ [ID08].	7 supporting 0 supporting 1 supporting	Try to implement both types of examples and aim for realistic ones.	
Humor	‘It mustn’t be forced. Discreetly funny but not too forced’ [ID04]. ‘In longer videos, it helps in any case, and, even in short videos, it does no harm. But as for whether it is necessary … [?]’ [ID10]. ‘I don’t really care. It must be interesting … but it doesn’t have to be done through humor … then better serious’ [ID03].	3 supporting 1 supporting 1 supporting	Design your videos to be appealing and use discreet humor.	Note: Spontaneous and self-ironic humor was designated an appropriate and pleasant form of humor.
Games (Quiz)	‘I am a very playful person, I’d like that’ [ID06]. ‘Yeah, you could do that, but you don’t need to’ [ID09]. ‘I find comparison with other students difficult’ [ID03].	7 supporting 1 supporting 0 supporting	Implement a quiz or games, if possible, consider offering the games as a voluntary option.	Ideally, the quizzes should run on mobile devices, too.
Videos as additional learning modality	‘It’s good to learn with such an additional offering [videos]. It is simply another way of learning’ [ID08].	5 supporting	Videos seem to be accepted as a learning tool.	

Note. Shaded rows mean that the category was defined deductively.

We interviewed a total of 11 participants (one student from each year [Years 1–6 in the German six-year curriculum], in addition to five students from Year 4). Eight participants were female and three male. This was equivalent to approximately 73% females and nearly coincided with the gender representation at RWTH Aachen’s faculty of medicine (69% male/31% female) [59]. Every potential participant who was asked to participate did so. No one dropped out.

We carried out face-to-face interviews in various locations to suit the participants (six in a social room at the faculty of medicine, four at home, and one in a café). Non-participants were present in the social room and in the café, but they were usually more than three meters away and out of auditory range. All the participants were medical students at RWTH Aachen University and aged 18–30 years. The participants’ academic performance ranged from sufficient to excellent. The average interview duration was 25.36 minutes (*SD* 7.59 min; median 23.12 min; range 15.58–36.67 min).

Because the last interviews conducted did not deliver new relevant information, we did not recruit more participants than the initially planned 11, following the concept of ‘theoretical saturation’ from grounded theory.

As intended, during the interview process, it was possible to discuss the reported characteristics in the literature as effective or presumably effective, thus achieving categorization, relativization, and consensus on the part of the interviewees as representatives of the respective stakeholders at the end-user level. Additionally, the spiral model enabled reflection on the temporally preceding interview content with the literature research findings and discussion of the results obtained with the subsequent interviewees. Thus, we obtained a broader basis for the development of recommendations than a literature review alone could have provided.

From the interview analysis, 10 deductively formulated categories and eight inductively found categories of content emerged (see Table 2).

Interestingly, various categories came up inductively, although we could have deductively set them because such recommendations appear in the literature. We considered these categories to be so self-evident that they did not need any discussion; however, we would have implemented them without further discourse due to their plausibility (e.g., ‘reduce extraneous processing,’ ‘structuring,’ and ‘chapters’). In our opinion, this underscores the perceived importance of these features and adds valuable focus for the further development of educational content for learners’ needs.

Most advice found in the literature search was confirmed through the interviews, and the students’ comments and clarifications enriched it. ‘Khan style’ (hand-written step-by-step explanations with voice commentary) and ‘talking head’ style (adding the speaker’s head to the visuals) were the only two areas in which the recommendations of the literature and the students’ opinions clearly differed. Our sample preferred subsequent text animations instead of the traditional Khan style.

Furthermore, the importance of take-home messages was outlined in the interviews. To our knowledge, however, this point is not a prominent recommendation in the literature.

Although the interviews mainly supported the previously found recommendations, they provided additional (specific) information about the students’ opinions and examples of what worked well in eLearning at our institution and what might work in various fields and situations. Therefore, the interviews helped accentuate the suggestions found in the literature.

Table 3 presents our conclusions, condensed into a single checklist for use in producing our videos (our checklist for educational videos for video-based eLearning). We identified 40 important points for well-designed educational videos and enriching techniques with concrete, processable questions to ensure the consideration of relevant design factors before creating content. Due to the various contexts of implementation, not every point must necessarily be respected in every video project.

**Table 3.** Our checklist for educational videos for video-based eLearning.

	Fulfillment? *
Does the content benefit from the “video” presentation mode (e.g., for teaching concepts)?	0 1 2 3
**Context**	
Is the content embedded in a larger context?	0 1 2 3
Are tangible examples (ideally chosen from everyday life and from specific [medical] situations) used to illustrate the content?	0 1 2 3
**Dual channel**	
Does the video provide audio and visuals that align with each other (“synchronizing/matching”)?	0 1 2 3
Does the video provide a continuous input flow (e.g., animations building up, Khan style)?	0 1 2 3
Are the visuals well-made and specifically appropriate for video use?	0 1 2 3
**Reduce extraneous processing**	0 1 2 3
Is the video clean and lacking in distracting elements (e.g., background music)?	0 1 2 3
Is unnecessary information absent from the video (“weeding”)?	0 1 2 3
Is redundancy avoided in the audio and visuals (e.g., the same text is not presented in spoken form and in an on-screen textbox)?	0 1 2 3
Are very important points highlighted (“signaling”)?	0 1 2 3
Are corresponding audio and visuals presented at the same time (“temporal continuity”)?	0 1 2 3
Are corresponding visuals presented near each other (“spatial continuity”)?	0 1 2 3
**Manage essential processing**	
Is the video length limited to about six minutes, or does it not exceed 10 minutes when the content cannot be presented didactically flawlessly in only six minutes?	0 1 2 3
Is the video divided into short chapters (“segmenting”)?	0 1 2 3
Does the video stop automatically after each chapter?	0 1 2 3
Does the audience have at least limited previous knowledge of the topic?	0 1 2 3
If not, is pre-training provided?	0 1 2 3
**Personalization**	
Is the audience addressed in a conversational style and/or an informal setting?	0 1 2 3
Does the narration feature simple syntax?	0 1 2 3
Is the speed appropriate (≥ 160 words per minute)?	0 1 2 3
Is the audience addressed directly so that it is clear that the video was made for its members?	0 1 2 3
Does a coherent structure exist throughout all videos (e.g., recognition patterns)?	0 1 2 3
Do take-home messages summarize the content at the end of the video?	0 1 2 3
Does the video include (light and self-deprecating) humor to an appropriate degree?	0 1 2 3
**Active learning**	
Does the video include guiding questions?	0 1 2 3
Are there questions and tests concerning the video to use during or after watching?	0 1 2 3
Is a player-vs.-player quiz implemented?	0 1 2 3
**Usability**	
Is the eLearning platform easy to use, structured, and visually appealing?	0 1 2 3
Is there an alternative presentation modality for users with visual disabilities (e.g., audio only)?	0 1 2 3
Are the users familiar with the platform?	0 1 2 3
Is the video tagged with keywords?	0 1 2 3
Is the platform enabled for mobile use?	0 1 2 3
Can the eLearning program be used “out-of-the-box” on any device?	0 1 2 3
Is the content available early so that users can prepare for the course when they have the time?	0 1 2 3
**Planning in advance**	
Is there a script for every video planned?	0 1 2 3
Is there a written summary for the users?	0 1 2 3
Is the video reusable, and does it exclude references to resources that you do not control (e.g., links)?	0 1 2 3
Is the video modular so that it is unnecessary to recreate it completely if anything changes?	0 1 2 3
**Administration**	
Does the platform allow monitoring to evaluate the use of the content?	0 1 2 3
Have all laws (e.g., copyright and data protection) been observed?	0 1 2 3
Has the video been reviewed and evaluated by a peer?	0 1 2 3

*Degree of fulfillment: not at all fulfilled (0), slightly fulfilled (1), moderately fulfilled (2), completely fulfilled (3).

## Discussion

Although eLearning and educational video are prominent topics in current research and university discourse, we were unable to find a comprehensive paper that considered most of the points relevant to our needs in relation to creating a video-based eLearning course in evidence-based medicine. Most of the recommendations we discussed can be found via a literature search, but to our knowledge, a broad compilation of these is novel.

Our work provides an extensive guide for video-based eLearning to meet the needs of teaching evidence-based medicine. In the context of the interviewed sample (consisting solely of medical students) and our focus on teaching the ‘statistical’ way of thinking (which is an unaccustomed concept for many medical students and is not integrated into the learners’ daily lives), the user value was reported to be highly appropriate for the targeted group. Nonetheless, our results should be applicable to other fields of (medical) education, especially where the aim is teaching conceptual knowledge and communicating ideas, concepts, and techniques for problem solving, as our research focus was applicable to such settings.

However, our work must be compared to articles that describe the development of eLearning offerings on a conceptual level (e.g., de Leeuw et al. [48]). Moreover, approaches such as the ‘Online Nursing Education Best Practices Guide’ by Authement and Dormire [47] should be mentioned. This approach provides an instructor checklist with 33 points focusing mainly on organizational issues for online education, which represents an important support for creating and conducting successful eLearning programs. Choe et al.’s ‘Summary of Best Practices for Creating Engagement in Educational Videos’ [30] is based on a text-based survey of undergraduate students. It describes and compares different video styles based on Mayer’s principles of multimedia learning, as well as students’ perceptions, and it can be a valuable aid for video creation. The aim of their work is close to our own, namely, to assist in quality educational video development, although it focuses on comparing different forms of presentation to derive valuable recommendations. However, the goal of their work was not to develop comprehensive guidelines for creating educational videos, and it included neither a systematic literature review nor consensus developed via interviews. Furthermore, we acknowledge studies such as that by Young et al. [29], who outlined principles for effective educational videos. Although the authors describe how their techniques are used for a specific topic, the beneficial general aspects are clearly identifiable. Moreover, the work of Roshier et al. [14] deserves mention. Based on focus group interviews and the authors’ experiences, it presents valuable suggestions for developing a video-based eLearning offering. Although the authors neither conducted a systematic literature review nor focused on educational theories, they provide orientating guidance that can contribute to the successful implementation of educational videos online.

General underlying models, such as the ADDIE model, which is based on generic analysis, design, development, implementation, and evaluation [20], and dividing video creation into preproduction, production, and postproduction stages [46], should also be mentioned. In the sense of the ADDIE model, our work enriches the design and development component, specifically for educational videos in teaching conceptual knowledge in medicine.

Although some findings overlap, our compilation of the requirements and recommendations in practicable guide form, informed by the available evidence and the findings of our qualitative research approach, enhances the existing literature at a new level by covering a broad spectrum of issues and providing concrete recommendations along with a checklist.

While our work and teaching aim relate to teaching concepts and knowledge but not (surgical) procedures, we excluded guidelines on surgical education videos from our literature search. Nevertheless, considering approaches from surgery might enhance the recommendations, particularly for situations where the teaching aim cannot be categorized as either conceptual knowledge or surgical procedures. Karic et al.’s [60] ‘Ideal Third Year Medical Student Educational Video Checklist’ mentions guidance and describes critical maneuvers, time efficiency, identifying instruments, and trocar placement as key aspects, among others. Apart from time efficiency, the ‘surgical setup’ is also described by Chauvet et al. [61] as a crucial point for creating effective surgical educational videos. Providing all the information necessary to teach vividly is also important in teaching concepts and educational video in general.

In our work, we encourage the enrichment of data collection with interviews to confirm suitability for individual situations. Although our sample size of interviews was modest (*n* = 11), the different points of view provided general considerations, further fields of interest, and specific comments on the appropriate form of video-based eLearning in our setting that may not have otherwise arisen. In addition, the confirmation and, sometimes, contextual relativization provided were relevant.

Interestingly, one point that was crucial to the interviewed students and, in our opinion, a relevant aspect of effective video design was the use of take-home messages. To our knowledge, this is not prominently recommended in the literature. This aspect certainly calls for further research.

However, given our results, one must keep in mind that good teaching always includes the individual component of the respective teacher, and the context and audience must always be considered. Thus, in some situations, a humorous approach may have a positive effect, whereas in others, it may be inappropriate (e.g., in some medical topics).

Furthermore, the potential gaps between ‘pleasing students,’ ‘perceived beneficial factors by students,’ and ‘fostering learning’ should be noted. Although the aim should be to maximize both learning and student satisfaction, particularly effective features for learning may be unpopular and vice versa. For instructional videos that follow Mayer’s principles, thanks to the data presented by Choe et al. [30], there is at least some indication that ‘pleasing students’ and ‘fostering learning’ need not be mutually exclusive, as evidenced by the similar effectiveness of the video styles examined. Therefore, one could fall back on features perceived by students as positive and satisfying. However, it is a limitation of this work that only characteristics derived from Mayer’s cognitive theory of multimedia learning were considered. Regarding other characteristics, further research, preferably randomized controlled trials, is necessary.

Regarding the evaluation issue, it should be noted that this cannot be concluded with the first review but must be continued on an ongoing basis. In this regard, continuous evaluation systems and measures should be established. If more than sporadic criticism arises, certain elements could be reconsidered and improved if necessary.

Regarding the methodology, the ability to substantiate any guideline recommendation with a clear body of studies is highly desirable. Preferably, a meta-analysis of multiple randomized controlled trials should be conducted, each examining only one feature, and recommendations should be developed from the resulting body of data. However, since the necessary studies do not exist on a sufficiently large scale and cannot be produced with reasonable effort by most research teams, our grounded theory approach is crucial to filling this gap.

It is important to note that the guidelines and checklist were compiled using grounded theory as a guiding framework; thus, the results should be formally considered interpretative and theories in progress, given grounded theory’s inherent methodology. However, we have mitigated this aspect as far as possible by incorporating established standards and applying the resulting defined methodology. Thanks to grounded theory as a guiding framework, we were able to combine, in a meaningful manner, accepted methods that, in themselves, would not be subject of this criticism. Thus, grounded theory fulfills an intermediary role, as this approach integrates existing evidence, classifies it, expands it through interviews, and thereby achieves categorization, relativization, and consensus. Thus, using our approach, we can deliver recommendations of higher value than expert opinions on their own.

Our approach offers creators a guide to designing an educational video set and/or reviewing one using our checklist for educational videos for video-based eLearning. We are aware that not every single point must be implemented in every context, but by referring to the literature in Table 1, a creator can check whether a characteristic mentioned applies to a given project. Creators should at least consider all the items and be able to justify why not all have been implemented.

In this context, it should be mentioned again that we elaborated our checklist for the transfer of conceptual knowledge so that for intended applications (e.g., skill transfer), practitioners can check whether all of the recommendations should be applied in a given setting in this way (e.g., the controversy about avoiding background music [50]).

Our guide can also be used as a valuable basis for further evaluation and description of implemented eLearning offerings. Using our checklist, important features can be evaluated to improve outcomes, and reporting gaps can be identified and resolved in the revision process. Thus, whether reported outcomes are influenced by unknown design aspects can be revealed. Practitioners do not always report how the videos used in evaluation trials were created [18], thus, we propose our checklist as a practical tool for reporting relevant design decisions in creating educational videos.

## Conclusion

We created a comprehensive guide for creating video-based eLearning offerings for teaching evidence-based medicine using a spiral model approach consisting of grounded theory methodology, a systematic literature search, and student interviews to reach a consensus. The 40-item checklist introduced can be useful for creating, reviewing, and reporting on educational videos, especially if they focus on teaching conceptual knowledge.

### Outlook

Based on our guide for educational video creation, we developed a video-based eLearning offering to address the abovementioned statistical illiteracy of medical students. Our procedure, its implementation, how we dealt with problems that arose, and the evaluation trial are planned to be reported in a further publication.

## Appendix

**Box 1:** Search strings used for the initial literature search

((((video OR educational video OR video-based eLearning OR video based learning OR video eLearning OR video learning OR medical video OR video tape recording[MeSH]) AND (online)) AND (medical students[MeSH] OR students[MeSH])) AND (educational technology[MeSH])) AND (guidelines as topic[MeSH])
(((video OR educational video OR video-based eLearning OR video based learning OR video eLearning OR video learning OR medical video) AND online) AND ((medical students[MeSH] OR students[MeSH]) AND ((educational technology[MeSH]) AND education) NOT game) NOT flipped classroom)
education AND checklist AND online AND (students OR undergraduate)
multimedia guidelines AND education
eLearning design
humor AND video AND education
video based learning
video eLearning
video learning

### Additional Information Regarding the COREQ-Checklist

#### Research Team and Reflexivity

The first author, a male 4^th^-year MD student aged 21 years, conducted the interviews. Prior to the interviews, the other author trained him via a supervision dialogue, reflection on the literature, and postprocessing.

All participants were personally known to the interviewer, and at least occasional personal contact between the interviewer and the participants existed independently of this study. The interviewer and the participants were also medical students at the same faculty and, at the very least, were acquainted. The participants knew that the study’s goal was to create a requirement analysis to improve the faculty’s eLearning program for evidence-based medicine (i.e., by implementing educational video).

### Interview Guide “Fighting Statistical Illiteracy” – What Form Should Successful Video eLearning Take?

Watch? □ Recorder? □ Writing materials? □

Welcome, gratitude, concern (=> title), consent to recording? Estimated timeframe: 30 minutes. Explaining procedure incl. subjectivity, open-minded; everything may be important!

Your benefit: chance to make an impact on this course; help improve the teaching and learning environment

=> start recording

As a student, you are a member of our target group; therefore, this interview has the sense of customer orientation!

*Vision/Warm up:*

Appealing & effective eLearning offerings on the topic of fighting statistical illiteracy; here, video-based

‘fighting statistical illiteracy’ particularly means studying comprehension and the correct handling of terms and values => creating the ability to interpret and self-confidently classify studies and results, also with regard to medical risk-communication

But: Not a statistics course (although content may overlap)

*Exploration of present situation:*

Huge spectrum of knowledge to be acquired with simultaneous danger of bulimia learning.

Nevertheless, evidence-based medicine topics are important for advanced semesters (e.g., research activities, how to write a paper) => our motivation for offering a voluntary evidence-based medicine course

What is your opinion concerning eLearning?

Talking about current eLearning at our institution (key facts): Our institutional eLearning, if any, often consists only of presentations and texts. Only a few videos are available, and they are usually not embedded in the main courses. Generally speaking, there are not many interactions.

=> Does that bother you? Would you like more (comprehensive) eLearning?

Keywords: eLearning in general, effectiveness, convenience, no travel time, …

*Exploration of future situation:*

What do you expect from successful eLearning? [our goal: crisp and brief, but effective]

Do you consider videos to be advisable for this purpose?

What do you wish for? What are must-haves and nice-to-haves for you? What must not appear?

What videos do you wish for? Let’s talk about animations and Khan style.

What do you think about examples in explanations? How many make it boring; when does it become boring? Do you prefer everyday-life examples or specific (medical) examples?

What do you think about games (like a quiz app for playing against other students) or interactions in general with other course mates in this context? Would you play educational games?

Let’s talk about learning controls, tests, and repeated testing!

What would you prioritize in order to use eLearning effortlessly?

Would you invest 10 to 15 minutes per week in such a course?

Do you think you would learn successfully from such a course?

Let’s talk about motivation. What do you think is more engaging: videos or scripts? Are 2 pages of script too much for one week?

[refer to requirement analysis]

Summary, feedback, gratitude => stop recording! Goodbye, outlook => course offering in summer semester, results of interviews will be available later.

Taking field notes □

### Coding guidelines for deductive categories concerning the analysis of the interviews

**Table:**

Category name	Nominal/Ordinal	Values	Definition/Basic encoding rules	Anchor Example	Further encoding rules/Depth
Experiences concerning video-based learning	Nominal	–	This category must be coded if the interviewee mentions any experience concerning video-based learning, including best-case and worst-case experiences.	‘And that’s when I really went under the covers because that was just badly presented and I just stopped after two videos because I just got the crisis. But if videos are well prepared and presented in a summarizing way, I think it would make a lot of sense.’	–
Experiences concerning text-based learning	Nominal	–	This category must be coded if the interviewee mentions any experience concerning text-based learning, including best-case and worst-case experiences.	‘Sometimes I find it more pleasant to have a text, because you can jump back and forth at your own pace.’	–
Khan style	Ordinal	C1 endorses Khan style C2 ambivalent about Khan style C3 opposes Khan style	This category must be coded if the interview content deals with Khan style in the sense of implementing step-by-step written explanations/development of content OR in the sense of subsequent text animations.	‘Take notes on a tablet and the integrate that in eLearning. Yes, that’s it.’	Respect all commentaries and opinions on Khan style. Code every reaction after the interviewer’s question about Khan style.
Animations	Ordinal	C1 endorses subsequent animations C2 ambivalent about subsequent animations C3 opposes subsequent animations	This category must be coded if the interviewee mentions aspects concerning subsequent animations.	‘It’s cool for schematics if they build up one after the other’	Respect all commentaries and opinions on animations. Code every reaction after the interviewer’s question about animations.
Medical examples vs. everyday-life examples	Ordinal	C1 endorses medical examples C2 ambivalent about medical or everyday-life examples C3 endorses everyday-life examples	This category must be coded if the interviewee mentions his/her opinion on examples in education OR mentions aspects concerning this topic.	‘[I’d appreciate] both; first, I would look at everyday life and then at what helps me later in my job’	Respect all commentaries and opinions on examples. Code every reaction after the interviewer’s question about examples.
Testing	Ordinal	C1 endorses repeated self-testing C2 ambivalent about repeated self-testing C3 opposes repeated self-testing	This category must be coded if the interviewee mentions his/her opinion on self-testing OR mentions aspects concerning this topic.	‘Well, I have to say that for me personally, I always perceived that [self-tests were] very helpful. You had the question, could immediately think about it, and then got the feedback directly.’	Respect all commentaries and opinions on self-testing OR testing in eLearning. Code every reaction after the interviewer’s question about examples.
Games (Quiz)	Ordinal	C1 endorses games in eLearning C2 ambivalent about games in eLearning C3 opposes games in eLearning	This category must be coded if the interviewee mentions his/her opinion on games in eLearning in general OR mentions aspects regarding player-vs.-player quizzes.	‘That might be a pretty good solution to make it a little more playful and less static. It might not be the way to teach it completely, but I can imagine it as a good start or for recapitulation.’	Respect all commentaries and opinions on games and quizzes OR games in eLearning. Code every reaction after the interviewer’s question about games.
Usability	Nominal	–	This category must be coded if the interviewee mentions anything on usability in eLearning.	‘I would appreciate one to have the system under control so that it doesn’t crash all the time.’	Respect all commentaries and opinions on (perceived) usability in eLearning, including but not limited to best-case and worst-case examples.
Video length	Nominal	–	This category must be coded if the interviewee mentions anything on preferred or opposed length of educative videos.	‘The maximum would be about 15 minutes because, beyond then, I would no longer be attentive. Five minutes is already a good length, but the videos must not be shorter. Something between 5 and 15 minutes.’	Respect all commentaries and opinions on the duration of education videos, including but not limited to best-case and worst-case examples and conditions that might affect the opinions.
Talking Head	Ordinal	C1 endorses ‘talking head’ in videos C2 ambivalent about ‘talking head’ in videos C3 opposes ‘talking head’ in videos	This category must be coded if the interviewee mentions anything about the lecturer’s/creator’s on-screen presence in educational video and his/her perception of this.	‘I don’t care to see the speaker – it can be good, but it doesn’t have to be’	Respect all commentaries and opinions on a ‘talking head’ in educational videos and conditions that might affect the opinions.

## References

[1] Gigerenzer G, Gaissmaier W, Kurz-Milcke E, et al. Helping doctors and patients make sense of health statistics. Psychol Sci Public Interest. 2007;8(2):53–21. doi: 10.1111/j.1539-6053.2008.00033.x 26161749

[2] Wegwarth O, Schwartz LM, Woloshin S, et al. Do physicians understand cancer screening statistics? A National Survey of primary care physicians in the United States. Ann internal med. 2012;156(5):340–U152. doi: 10.7326/0003-4819-156-5-201203060-00005 22393129

[3] Ahmadi S-F, Baradaran HR, Ahmadi E. Effectiveness of teaching evidence-based medicine to undergraduate medical students: A BEME systematic review. Med Teach. 2015;37(1):21–30. doi: 10.3109/0142159X.2014.971724 25401408

[4] Weberschock T, Sorinola O, Thangaratinam S, et al. How to confidently teach EBM on foot: development and evaluation of a web-based e-learning course. Evid Based Med. 2013;18(5):170–172. doi: 10.1136/eb-2012-100801 22864372

[5] Vaona A, Banzi R, Kwag KH, et al. E-learning for health professionals. Cochrane Database Syst Rev. 2018;2018(8). CD011736. doi: 10.1002/14651858.CD011736.pub2 PMC6491176 29355907

[6] Chi DL, Pickrell JE, Riedy CA. Student learning outcomes associated with video vs. paper cases in a public health dentistry course. J Dent Educ. 2014;78(1):24–30. doi: 10.1002/j.0022-0337.2014.78.1.tb05653.x 24385521

[7] Murthykumar K, Veeraiyan DN, Prasad P. Impact of video based learning on the perfomance of post graduate students in biostatistics: a retrospective study. J Clin Diagn Res. 2015;9(12):ZC51–3. doi: 10.7860/JCDR/2015/15675.7004 PMC4717794 26813422

[8] Stockwell BR, Stockwell MS, Cennamo M, et al. Blended learning improves science education. Cell. 2015;162(5):933–936. doi: 10.1016/j.cell.2015.08.009 26317458

[9] Guo PJ, Kim J, Rubin R. How video production affects student engagement, In Proceedings of the first ACM conference on Learning @ scale conference - L@S ’14; Atlanta, Georgia, USA. 2014. p. 41–50.

[10] Mayer RE. Applying the science of learning: evidence-based principles for the design of multimedia instruction. Am Psychol. 2008;63(8):760–769. doi: 10.1037/0003-066X.63.8.760 19014238

[11] Mayer RE. Applying the science of learning to medical education. Med Educ. 2010;44(6):543–549. doi: 10.1111/j.1365-2923.2010.03624.x 20604850

[12] Yue C, Kim J, Ogawa R, et al. Applying the cognitive theory of multimedia learning: an analysis of medical animations. Med Educ. 2013;47(4):375–387. doi: 10.1111/medu.12090 23488757

[13] Lehmann R, Seitz A, Bosse HM, et al. Student perceptions of a video-based blended learning approach for improving pediatric physical examination skills. Ann Anat. 2016;208:179–182. doi: 10.1016/j.aanat.2016.05.009 27328405

[14] Roshier AL, Foster N, Jones MA. Veterinary students’ usage and perception of video teaching resources. BMC Med Educ. 2011;11(1):1. doi: 10.1186/1472-6920-11-1 21219639 PMC3025976

[15] Brockfeld T, Muller B, de Laffolie J. Video versus live lecture courses: a comparative evaluation of lecture types and results. Med Educ Online. 2018;23(1):1555434. doi: 10.1080/10872981.2018.1555434 30560721 PMC6300084

[16] Higgins Joyce A, Raman M, Beaumont JL, et al. A survey comparison of educational interventions for teaching pneumatic otoscopy to medical students. BMC Med Educ. 2019;19(1):79. doi: 10.1186/s12909-019-1507-0 30866922 PMC6417091

[17] Suppan M, Stuby L, Carrera E, et al. Asynchronous Distance Learning of the National Institutes of Health stroke scale during the COVID-19 pandemic (E-Learning vs video): randomized controlled trial. J Med Internet Res. 2021;23(1):e23594. doi: 10.2196/23594 33428581 PMC7812917

[18] Yiu SHM, Spacek AM, Pageau PG, et al. Dissecting the contemporary clerkship: theory-based educational trial of videos versus lectures in medical student education. AEM Educ Train. 2020;4(1):10–17. doi: 10.1002/aet2.10370 31989065 PMC6965666

[19] Frankl SE, Joshi A, Onorato S, et al. Preparing future doctors for telemedicine: an asynchronous curriculum for medical students implemented during the COVID-19 pandemic. Acad Med. 2021;96(12):1696–1701. doi: 10.1097/ACM.0000000000004260 34323861 PMC8603440

[20] Allen WC. Overview and Evolution of the ADDIE Training System. Adv Dev Hum Resour. 2006;8(4):430–441. doi: 10.1177/1523422306292942

[21] Overbaugh RC. Research-based guidelines for computer-based instruction development. J Res Comput Educ. 1994;27(1):29–47. doi: 10.1080/08886504.1994.10782114

[22] Mayer R, Moreno R. Nine ways to reduce cognitive load in multimedia learning. Educ Psychol. 2003;38(1):43–52. doi: 10.1207/S15326985EP3801_6

[23] Sweller J. Implications of cognitive load theory for multimedia learning. In: Mayer RE, editor The Cambridge handbook of multimedia learning. Cambridge: Cambridge Univ. Press; 2005. pp. 19–30.

[24] Brame CJ. Effective educational videos: principles and guidelines for maximizing Student learning from video content. CBE Life Sci Educ. 2016;15(4):es6. doi: 10.1187/cbe.16-03-0125 27789532 PMC5132380

[25] Dong C, Goh PS. Twelve tips for the effective use of videos in medical education. Med Teach. 2015;37(2):140–145. doi: 10.3109/0142159X.2014.943709 25110154

[26] Norman MK. Twelve tips for reducing production time and increasing long-term usability of instructional video. Med Teach. 2017;39(8):808–812. doi: 10.1080/0142159X.2017.1322190 28485662 PMC6262844

[27] Rana J, Besche H, Cockrill B. Twelve tips for the production of digital chalk-talk videos. Med Teach. 2017;39(6):653–659. doi: 10.1080/0142159X.2017.1302081 28332912

[28] Iorio-Morin C, Brisebois S, Becotte A, et al. Improving the pedagogical effectiveness of medical videos. J Vis Commun Med. 2017;40(3):96–100. doi: 10.1080/17453054.2017.1366826 28925762

[29] Young TP, Guptill M, Thomas T, et al. Effective educational videos in emergency medicine. AEM Educ Train. 2018;2(Suppl 1):S17–S24. doi: 10.1002/aet2.10210 30607375 PMC6304276

[30] Choe RC, Scuric Z, Eshkol E, et al. Student satisfaction and learning outcomes in asynchronous online lecture videos. CBE Life Sci Educ. 2019;18(4):r55. doi: 10.1187/cbe.18-08-0171 PMC6829069 31675279

[31] Raymond M, Iliffe S, Pickett J. Checklists to evaluate an e-learning resource. Educ Prim Care. 2012;23(6):458–459. doi: 10.1080/14739879.2012.11494162 23232142

[32] Corbin JM, Strauss A. Grounded theory research: Procedures, canons, and evaluative criteria. Qual Sociol. 1990;13(1):3–21. doi: 10.1007/BF00988593

[33] Strauss A, Corbin JM. Grounded theory methodology: an overview. In: Denzin NK, and Lincoln YS, editors. Handbook of qualitative research. Thousand Oaks, USA: Sage Publications; 1994. p. 273–285.

[34] Strübing J. Grounded Theory. In: Bohnsack R, Flick U, Lüders C, editors. Qualitative Sozialforschung. Wiesbaden: Springer VS; 2014.

[35] Mayring P. Qualitative content analysis: theoretical foundation, basic procedures and software solution. Klagenfurt, Austria: Klagenfurt; 2014. p. 143.

[36] Mayring P. Qualitative Inhaltsanalyse: Grundlagen und Techniken. 12th ed. Weinheim, Germany & Basel, Switzerland: Beltz; 2015.

[37] Chen Y, Yang K, Marušic A, et al. A reporting tool for practice guidelines in health care: the RIGHT statement. Ann Intern Med. 2017;166(2):128–132. doi: 10.7326/M16-1565 27893062

[38] Chen Y, Yang K, Marusic A, et al. RIGHT Explanation and Elaboration: guidance for reporting practice guidelines. 2017.

[39] Brouwers MC, Kho ME, Browman GP, et al. AGREE II: advancing guideline development, reporting and evaluation in health care. CanMed Assoc J. 2010;182(18):E839–42. doi: 10.1503/cmaj.090449 PMC3001530 20603348

[40] Brouwers MC, Kerkvliet K, Spithoff K. The AGREE Reporting Checklist: a tool to improve reporting of clinical practice guidelines. BMJ. 2016;352:i1152. doi: 10.1136/bmj.i1152 26957104 PMC5118873

[41] AGREE Next Steps Consortium. The AGREE II Instrument [Electronic version]. 2017.

[42] Moher D, Liberati A, Tetzlaff J, et al. Preferred reporting items for systematic reviews and meta-analyses: the PRISMA statement. PLoS Med. 2009;6(7):e1000097. doi: 10.1371/journal.pmed.1000097 19621072 PMC2707599

[43] Tong A, Sainsbury P, Craig J. Consolidated criteria for reporting qualitative research (COREQ): a 32-item checklist for interviews and focus groups. Int J Qual Health Care. 2007;19(6):349–357. doi: 10.1093/intqhc/mzm042 17872937

[44] Sait S, Tombs M. Teaching medical students how to interpret chest X-Rays: the design and development of an e-Learning Resource. Adv Med Educ Pract. 2021;12:123–132. doi: 10.2147/AMEP.S280941 33574725 PMC7872944

[45] Mulcahy RS. Creating effective eLearning to help drive change. ACS Chem Health Saf. 2020;27(6):362–368. doi: 10.1021/acs.chas.0c00091 34191965

[46] Fleming SE, Reynolds J, Wallace B. Lights… camera… action! a guide for creating a DVD/video. Nurse Educ. 2009;34(3):118–121. doi: 10.1097/NNE.0b013e3181a0270e 19412052

[47] Authement RS, Dormire SL. Introduction to the online nursing education best practices guide. Sage Open Nurs. 2020;6:2377960820937290. doi: 10.1177/2377960820937290 33415291 PMC7774434

[48] de Leeuw R, Scheele F, Walsh K, et al. A 9-step theory- and evidence-based postgraduate medical digital education development model: empirical development and validation. JMIR Med Educ. 2019;5(2):e13004. doi: 10.2196/13004 31333194 PMC6876560

[49] Cross A, Bayyapunedi M, Cutrell E, et al. Combining the benefits of handwriting and typeface in online educational videos. CHI 2013. 2013.

[50] Wong G, Apthorpe HC, Ruiz K, et al. An innovative educational approach in using instructional videos to teach dental local anaesthetic skills. Eur J Dent Educ. 2019;23(1):28–34. doi: 10.1111/eje.12382 30069994

[51] Maloy J, Fries L, Laski F, et al. Seductive details in the flipped classroom: the impact of interesting but educationally irrelevant information on Student learning and motivation. CBE Life Sci Educ. 2019;18(3):r42. doi: 10.1187/cbe.19-01-0004 PMC6755318 31469621

[52] Spreckelsen C, Juenger J. Repeated testing improves achievement in a blended learning approach for risk competence training of medical students: results of a randomized controlled trial. BMC Med Educ. 2017;17(1):177. doi: 10.1186/s12909-017-1016-y 28950855 PMC5615441

[53] McCoy L, Lewis JH, Dalton D. Gamification and multimedia for medical education: a landscape review. J Am Osteopath Assoc. 2016;116(1):22–34. doi: 10.7556/jaoa.2016.003 26745561

[54] Adam M, McMahon SA, Prober C, et al. Human-centered design of video-based health education: an iterative, collaborative, community-based approach. J Med Internet Res. 2019;21(1):e12128. doi: 10.2196/12128 30698531 PMC6372941

[55] Phelan J. Ten tweaks that can improve your teaching. Am Biol Teach. 2016;78(9):725–732. doi: 10.1525/abt.2016.78.9.725

[56] Jang HW, Kim KJ. Use of online clinical videos for clinical skills training for medical students: benefits and challenges. BMC Med Educ. 2014;14(1):56. doi: 10.1186/1472-6920-14-56 24650290 PMC3994418

[57] Margolis A, Porter A, Pitterle M. Best practices for use of blended learning. Am J Pharm Educ. 2017;81(3):81(3. doi: 10.5688/ajpe81349 PMC5423065 28496269

[58] Yavner SD, Pusic MV, Kalet AL, et al. Twelve tips for improving the effectiveness of web-based multimedia instruction for clinical learners. Med Teach. 2015;37(3):239–244. doi: 10.3109/0142159X.2014.933202 25109353

[59] RWTH Aachen University, *Zahlenspiegel 2017*. 2018.

[60] Karic B, Moino V, Nolin A, et al. Evaluation of surgical educational videos available for third year medical students. Med Educ Online. 2020;25(1):1714197. doi: 10.1080/10872981.2020.1714197 31920174 PMC6968551

[61] Chauvet P, Botchorishvili R, Curinier S, et al. What is a good teaching video? Results of an online international survey. J Minim Invasive Gynecol. 2020;27(3):738–747. doi: 10.1016/j.jmig.2019.05.023 31233782

